# Black, Asian and Minority Ethnic men’s experiences of gender-based violence, help-seeking behaviours and psychosocial interventions in the United Kingdom: a systematic review

**DOI:** 10.3389/fpubh.2026.1695675

**Published:** 2026-03-13

**Authors:** Tarela Juliet Ike, Dung Ezekiel Jidong, Nikki Carthy, Chiyem Lucky Nwanzu, Laura Gair, Callistar Kidochukwu Obi, Bernard Ozofere Ishioro, Mieyebi Lawrence Ike, Evangelyn Ebi Ayobi, Peremi Richmond Ike, Tariq Mahmood, Rukevwe Francis Doghor

**Affiliations:** 1 School of Social Sciences, Humanities and Law, Department of Law, Policing and Investigation, Teesside University, Middlesbrough, United Kingdom; 2 Division of Psychology and Mental Health, University of Manchester, Manchester, United Kingdom; 3 School of Social Sciences, Humanities and Law, Department of Psychology, Teesside University, Middlesbrough, United Kingdom; 4 Department of Psychology, Delta State University, Abraka, Nigeria; 5 Department of Economics, Faculty of the Social Sciences, Delta State University, Abraka, Nigeria; 6 Western Governors University, Millcreek, UT, United States; 7 Manchester Global Foundation, Manchester, United Kingdom; 8 Tare Wyd Legal Chambers, Warri, Nigeria; 9 Arooj, Bradford, United Kingdom; 10 Beryl Foundation, London, United Kingdom

**Keywords:** ethnic minority men, gender-based violence, help-seeking, psychosocial intervention, United Kingdom

## Abstract

**Background:**

Gender-Based Violence (GBV) is an issue of public health concern. Yet most research on GBV focuses predominantly on women. A gap remains in a review of Black Asian and Minority Ethnic (BAME) men’s experiences of gender-based violence, help-seeking behaviours, and interventions to improve their psychosocial wellbeing in the United Kingdom. This review addressed the gap.

**Methods:**

Six databases were searched (PsycINFO, ProQuest Central, Scopus, Applied Social Sciences Index and Abstract, PudMed, and Embase) for published articles between 2011 and June 2025. *N* = 10 studies met the inclusion criteria. The relevant data were synthesised and thematically analysed.

**Findings:**

The review found that sexual and physical abuse, masculinity, and societal perceptions of men as abusers pose barriers to help-seeking. Religio-cultural factors, including psychological effects, victimisation from service providers, also limit help-seeking. It also found that there was persistent recourse to informal platforms for support alongside limited psychosocial interventions.

**Conclusion:**

Culturally adapted psychosocial interventions are suggested alongside testing using randomised controlled trials.

**Systematic review registration:**

https://www.crd.york.ac.uk/PROSPERO/view/CRD420261280683, identifier PROSPERO (CRD420261280683).

## Introduction

Gender-Based Violence (GBV) raises significant public health concerns, with men adversely affected. Data from the Mankind initiative (1), which is the largest third sector organisation providing support for males affected by GBV, indicated that in the United Kingdom (UK), one in five men, equating to 21.7% (5.1 million men), have been a victim of domestic abuse in their lifetime. The Office for National Statistics (2) data indicate that 21% of male victims between the period of 2022 and 2023 failed to disclose that they were a victim of partner abuse to anyone, and 6.5% of males, in comparison to 2.8% of women, indicated considering committing suicide due to experiencing partner abuse. Of central concern is Black, Asian, and Minority Ethnic (BAME) males’ experiences of gender-based violence, help-seeking behaviour, and intervention in the UK. Studies have shown that BAME males face challenges and intersecting inequalities in the UK (3–5).

The challenges BAME men face includes disproportionate stop and search of minoritised males compared to the white males (3), higher risk of being compulsorily admitted under the Mental Health Act 1983 and 2007 (4), poorer access to mental health services (5), and mistrust for mental health services (6). Other challenges include perceived discrimination by health services providers (7), the criminal justice system (8), and education for minoritised populations who are at a higher risk of exclusion from schools in the UK (9, 53). The intersecting challenges further serve to marginalise BAME men in the UK. For example, a study by the University of Cambridge (10) with over 10,000 black males across the UK indicates that an estimated 87% of respondents distrust the criminal justice system and also expect to receive poor levels of health care due to their race, while 95% indicate the UK curriculum appears not to reflect black experiences. BAME men are less likely to be believed for experiencing gender-based violence (11, 12). Yet, as the preceding literature shows, there remains a gap to address this urgent problem for minoritised males in the UK. Anchored in Crenshaw’s (13) intersectionality theory, the review makes an original and significant contribution by addressing the gap in the synthesis of literature on BAME men’s experiences of gender-based violence, help-seeking, and interventions to improve their psychosocial wellbeing.

A growing body of literature has tended to address the adverse impact of gender-based violence on men and their poor help-seeking behaviour. In the global north, factors such as harmful gender specific stereotypes on sexuality and masculinity often serve as barriers to help-seeking behaviours (14, 48). Other barriers include fear of being disbelieved, feelings of guilt or shame (15), low self-esteem, fear of societal reprisal or being taken less seriously by service providers (Bates, 2020) (12) and male victimisation (16). For example, Carthy et al. (16) study, comprising 12 mid-lifers and older men, found, among others, that men faced discrimination from the legal system and the perceived police extensive support for women with less emphasis on men. Previous studies also show that it often takes years following experiencing sexual abuse for men to disclose such experiences (17). In the global south, other studies in countries like South Africa have focused on men’s silence in reporting gender-based violence, stigma suffered, and the fear of being ridiculed (18). Issues such as shame and socio-cultural barriers limit the help-seeking behaviours of affected men (19). Another study in Nigeria emphasised the role of social media in framing and reinforcing harmful stereotypes, gender inequalities and marginalised males affected, as such, exacerbating the culture of silence and poor help-seeking among male victims (20).

While the preceding literature offers valuable insights, there remains a significant gap in understanding the victimisation of BAME men. Despite growing recognition of intersectionality, the unique experiences of BAME victims—particularly in the contexts of abuse, trauma, and marginalisation—are often overlooked as most studies tend to focus on females (21–23). As Crenshaw’s (13) intersectionality theory posits, factors such as class, race, and gender, as social categorisations, often intersect and combine to create experiences of discrimination for the affected population. BAME males face myriad challenges, including cultural barriers surrounding victimhood (Bates, 2020) (24) and limited recognition of their experiences within support services, and systemic barriers to accessing health care (5). Our review thus addresses the gap to arrive at recommendations to inform policies in understanding BAME men’s experience, their help-seeking behaviours and intervention, including what works and what does not to improve the psychosocial well-being of men affected by gender-based violence. Hence, the research question:

What are Black, Asian and Minority Ethnic (BAME) men’s experiences of gender-based violence, help-seeking behaviours, and interventions to improve their psychosocial wellbeing in the UK?

In this review, the definition of Gender-based Violence (GBV) by the European Commission is adopted which defined it as:

“Violence directed against a person because of that person’s gender or violence that affects persons of a particular gender disproportionately. […] It can include violence against women, domestic violence against women, men or children living in the same domestic unit. Although women and girls are the main victims of GBV, it also causes severe harm to families and communities.”

The rationale for adopting the definition is its acknowledgement of the victims of gender-based violence regardless of gender, alongside the form of GBV, including sexual or physical violence and psychological abuse. Gender-based violence also includes domestic violence, sex-based harassment, forced marriage, or online violence (54). To address the review question, the next section reports the methodology adopted in the review. The findings and discussion are presented alongside the conclusion and recommendations.

## Method

### Protocol/search strategy

The review protocol adopted the Preferred Reporting Items for Systematic Reviews and Meta-Analyses (PRISMA). The PRISMA guidelines help ensure the replication, rigour, and transparent reporting of the systematic review process (47). The protocol was also designed to clearly specify the review’s inclusion and exclusion criteria, as well as the analysis method, before undertaking the review (49). In line with global best practices, the review protocol was registered with the PROSPERO Registry (no. CRD420251078854). Ethical approval was not sought for the review, as all studies relied on are in the public domain and ethical approval is not required to conduct a systematic review.

### Study’s inclusion and exclusion criteria

Our review purpose was to synthesise Black, Asian and Minority Ethnic (BAME) Men’s experiences of gender-based violence, help-seeking behaviours and interventions to improve their psychosocial wellbeing in the UK. The review’s search terms were therefore designed to yield a robust body of literature relevant to the review. The inclusion criteria for the review are: (i) studies including BAME males with lived experiences, or perspective on gender-based violence or help-seeking in the UK; (ii) studies which tested interventions such as psychosocial interventions, educational programmes, mindfulness interventions, school based interventions, Cognitive Behaviour Therapy, faith-based intervention, orientations or any psychosocial programme as appropriate aimed at addressing gender-based violence or their help-seeking behaviours for BAME male affected by gender-based violence; (iii) studies using either quantitative, qualitative, mixed methods, randomised control trials, cohort study, longitudinal study, or quasi experimental design or study with pre or post-intervention methods evaluating BAME men’s experiences or perspectives of the effectiveness of gender-based violence interventions, including help seeking behaviours. (iv) Studies focusing on gender-based violence and help-seeking behaviour will be included if BAME men with lived experiences are included in the population and the study is based in the UK. (v) Studies focusing on BAME males’ experiences of gender-based violence or help-seeking behaviour or interventions across the life course, or where BAME males are included as part of the population.

The review exclusion criteria are: (i) Studies not focusing on experiences or interventions to address gender-based violence or help-seeking behaviour, and which did not include the BAME men’s population in the UK. (ii) The studies addressed interventions that were not related to gender-based violence or experiences and improving the mental health or help-seeking behaviour and or social well-being for the BAME population in the UK; (iii) The study did not include BAME males population and was not based in the UK (iv) studies not in English language or where there is absence of English language translation if written or delivered in any other language(s); and (v) conference proceedings, grey literature, or studies that did not publish empirical data.

### Search strategy

The review adopted the PICo model, including Population (BAME males in the UK), the phenomenon of Interest (gender-based violence, help-seeking, and domestic violence interventions), and Context (gender-based violence among BAME males, their lived experiences, and interventions designed to address the problem; (50)). The design of strategic search terms was informed by Boolean operators (AND/OR/NOT) to ensure rigour (51). The search terms adopted are included and listed in the Table 1.

**Table 1:** Search terms adopted for the review.

Search terms	“BAME males” AND “violence” OR “abuse” AND “mental health” AND “social wellbeing” AND “UK.” “Interventions” AND “gender-based violence” AND “UK,” “Caribbean male” AND “gender-based violence” AND “CBT” AND “mental health” in UK,” “BAME male” OR “older adult male” AND “children” AND “intervention” AND “domestic violence in UK.” “Black Men” OR “South Asian men” AND “Gender-based violence” OR “educational intervention” AND “intervention” AND “addressing domestic violence in the UK.” “Black male” NOT “female,” OR “Canada” OR “Asia” OR “USA” AND “programmes addressing domestic violence.” “Africa” OR “Afro-Caribbean” OR “Black Caribbean” OR “Caribbean men” AND “gender-based violence” OR “intimate partner violence” OR “domestic violence” OR “intimate partner violence” OR “partner abuse” OR “sexual violence” OR “coercive control” OR “interpersonal violence” AND “African diaspora” OR “Caribbean diaspora” OR “United Kingdom” OR “Scotland” OR “England” OR “Wales” OR “Northern Ireland.”

The search terms were used to search six databases, including PsycINFO, ProQuest Central, Scopus, Applied Social Science Index and Abstract, PubMed, and Embase. To supplement our review searches and ensure that no studies were missed, the reference lists of papers meeting the inclusion criteria were manually searched. Searches of published articles in University libraries (e.g., the first author’s university affiliation) were also conducted. The review synthesised studies published between 2011 and June 2025 to maintain currency and relevance.

### Included studies screening and selection

**Figure 1:**
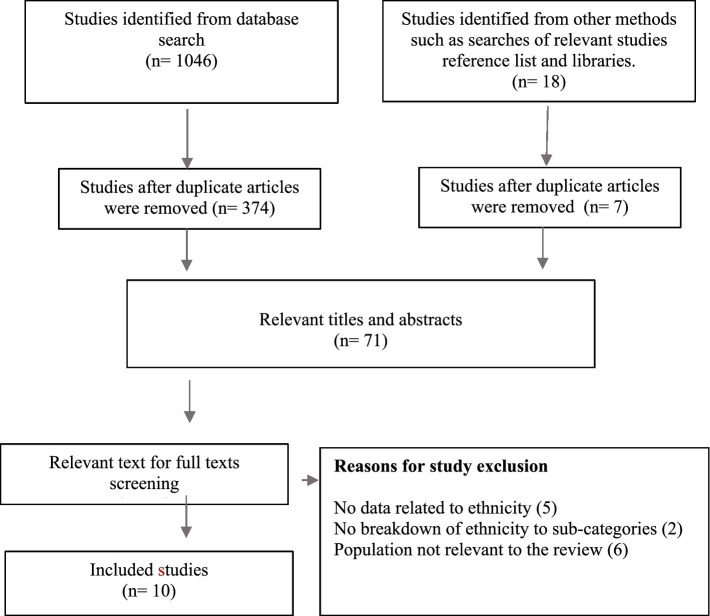
PRISMA flow diagram showing trajectory of the literature review process.

Based on the databases searched, a total of 1,046 records were identified. Of the 1,046 studies, (*n* = 683) were duplicates and were removed. The remaining studies’ titles and abstracts were screened by three members of the review team using the study’s inclusion and exclusion criteria. Studies that did not meet the inclusion criteria were excluded from the review. In total, 10 studies met the review inclusion criteria (See Figure 1, which illustrates the flow diagram of studies and the final inclusion of relevant studies meeting the review criteria). Three reviewers further evaluated the relevant studies to ensure they met the criteria. Where discrepancies existed, they were addressed through consultation and discussion among the review team.

### Assessment of risk of bias

To ensure rigour, for qualitative studies, the Joanna Briggs Institute (JBI) Critical Appraisal Checklist for Qualitative Research was used to assess these studies. It comprises a 12-item checklist to aid critical assessment. Notable questions contained in the JBI checklist included:

Are participants and their voices adequately represented? Is there congruity between the research methodology and the interpretation of results?. Assessment was conducted, and reporting used Yes/No/Unclear/Not applicable, as provided in the JBI checklist. In mixed-method studies, the Critical Appraisal Skills Programme (55) was used to aid assessment. The CASP is a 10-question checklist of quality criteria that aids in the systematic evaluation of studies that meet the review’s inclusion criteria. The CASP also includes questions such as: Is a qualitative/ quantitative method appropriate? And was the data collected in a way that addressed the research issues?. A “yes,” “no,” or “cannot tell” option was provided in the CASP checklist for most questions, along with spaces to record the reasons for the answers. The CASP also provides criteria to aid assessment for the potential risk of bias in the qualitative aspect of mixed-method studies. Collectively, the studies in our review highlighted a low risk of bias (see Appendix Table 2 for studies assessed using CASP). The JBI Critical Appraisal Checklist for Randomised Controlled Trials, comprising a 14-item checklist, was initially intended to be used to critically evaluate studies using the randomised controlled trial (RCT) design; however, no studies adopted the RCT design. See the Appendix for all studies’ risk of bias assessments, conducted in line with the relevant checklist as appropriate. Three reviewers assessed each study’s risk of bias. Where discrepancies existed, this was resolved in consultation with the wider research team.

## Data synthesis

The key findings emanating from the included studies were thematically synthesised to answer the study’s research question. The thematic analysis of the synthesised study was also informed by the conceptual framework (25). This involves an initial conceptual structuring of the review informed by a guiding theory (13) alongside a description of how the review was conducted and a critical analysis of each study (25). The synthesis matrix facilitated the critical evaluation and analysis of each included study, highlighting its weaknesses, strengths, and gaps. In conducting the synthesis, we adopted a four-stage approach. This includes (i) implementation of the appropriate and relevant literature search; (ii) identification of the key elements and ideas; (iii) organisation of the main ideas and key elements of the studies; (iv) synthesis of existing data and the development of the need for a new research intervention. The synthesis matrix aids our organisation of research sources in a Word document, mapping included studies against key themes and ideas to visually identify patterns, gaps, and debates in the literature. It also aided in sorting and categorising the themes that emerged from the synthesised included studies. To ensure rigour, an ‘index card method’ was also adopted in stage four to identify and organise the key elements and ideas from stages ii and iii. The index card method was applied in the current review by summarising the key points from included sources onto individual cards, categorising the points by theme, and arranging them to build a visual map, which aided in highlighting connections and gaps that informed the writing of the findings.

## Findings

Based on the thematic synthesis of relevant studies, the following themes emerged, including (i) sexual and physical abuse, (ii) masculinity, (iii) societal perceptions of men as abusers and barriers to help-seeking, (iv) religio-cultural factors, (v) psychological effect, (vi) victimisation from service providers as barriers to help seeking, (vii) resort to informal platform for support and (viii) psychosocial interventions. Table 2 is summarising each theme and corresponding studies.

**Table 2:** Each theme and its alignment with the studies.

S/n	Themes	Studies
i	Sexual and physical abuse	(11, 26, 27, 28, 29, 30, 31)
ii	Masculinity	(11, 12, 24, 28, 32)
iii	Societal perceptions of men as abusers and barriers to help-seeking	(11, 12, 24, 32)
iv	Religio-cultural factors	(11, 12, 24, 28, 32)
v	Psychological effect	(11, 12, 24, 28, 31, 32)
vi	Victimisation from service providers as barriers to help seeking	(11, 12)
vii	Resort to informal platform for support	(11, 12, 28, 32)
viii	Psychosocial interventions	(12, 28, 32)

The themes are reported below.

i Sexual and physical abuse

A notable pattern in the synthesised study is the myriad forms of abuse men faced across the life course. This comprises child sexual abuse (26–28), sexual abuse of adolescents (29) and adult men (30), including physical and emotional abuse (11, 31). The effect of sexual abuse was reported to significantly affect males, with some having criminal records (27), others engaging in drug use and further experiencing homophobia and racism (52). Jaspal et al.’s study, comprising 432 black and minority ethnic males, found that men with a history of sexual abuse reported a higher rate of drug use compared to those with no history of sexual abuse (52). The affected males were also found to engage in sexual risk, such as HIV risky sexual behaviour, which entails engaging in sexual activities that significantly increase the chance of acquiring or transmitting HIV through unprotected sex with partners whose HIV status is positive or unknown (52).

ii Masculinity

A recurring pattern across the synthesised studies was the perceived masculine expectations society ascribes to men. In this light, men reported feeling reluctant to seek help for fear of being disbelieved or the shame that comes with being a victim of gender-based violence (11, 12, 24, 28, 32). This is further compounded by victimisation, which the men often perceived to be interpreted by society as being associated with a failure to hold on to masculine expectations and exhibition of weakness (12). A study by Hogan et al. (32) comprising 26 participants highlighted how affected male participants were reluctant to accept they were victims due to the perceived sense that it was emasculating, and as a result, they avoided seeking help to prevent shame. The preceding studies thus highlight how the intersection of masculinity and males’ gendered expectations ascribed by socio-cultural norms further marginalised the affected males, with implications for limiting their help-seeking behaviours.

iii Societal perceptions of men as abusers and barriers to help-seeking

Several studies highlighted the role of societal perception, which construe men as the abusers, not one to be subject to abuse or one not in a position to be hit by a woman as exacerbating their risk of experiencing abuse (11, 12, 24, 32). Other studies further highlighted the role of the media in exacerbating the negative stereotype against males as the abuser (11). In addition, other factors such as strength and physical size differences between the male and female ignite victims’ fear that they will not be believed by society to be abused. Hogan, Clarke & Ward's (32) highlighted how males are susceptible to violence due to societal perceptions of masculine norms, which sees the normalisation of violence by men against men but frowns upon any physical force against women due to its inconsistency with moral norms and expectations. Myrie & Schwab's (24) study found that when black males face abuse, it affects their confidence, leading to low self-esteem and a sense of self.

iv Religio-cultural factors

Some studies reported that religion and cultural factors contribute to the negative stereotypes men experience (11, 12, 24, 28, 32). Religious and cultural factors were often construed as reasons for remaining in the abusive relationship and not leaving (11). For example, in Bates' (11) study, respondents reported church doctrine on marriage as something lasting forever as the reason for not leaving. Another study cited cultural expectations to endure and prevent embarrassment, especially when children are involved, as the barriers limiting help-seeking behaviour or even leaving the abusive relationship (11). One study focusing on South Asian men’s experiences of child sexual abuse in the UK found that the expectation to live up to cultural ideals and obligations, coupled with the need to keep everyone happy, limited the affected males’ ability to speak up for fear of letting everyone down (28).

v Psychological effect

A number of studies reported the psychological impact gender-based violence had on the affected males and this spans from psychological effect such as post-traumatic stress disorders (12, 24, 28, 31), depression, anxiety, suicidal ideation, and crying to physical violence experienced (11, 32). Bates' (11) study, comprising 161 participants with lived experiences, found among others that some participants expressed intention to kill themselves using sleeping pills just to get over the abuse (12). Hester et al.'s (31) study, comprising 1,403 patients, also found that men experiencing and perpetrating domestic violence and abuse were more likely to have anxiety and depression.

vi Victimisation from service providers as barriers to help seeking

Some studies highlighted the role of formal service provider victimisation of the affected male through their responses as barriers to help seeking (11, 12). Some participants highlighted the issue of being blamed following a call to the help line, with others reporting being ridiculed and laughed at by the police who appeared not to believe the male victim who reported (11, 12). The perceived victimisation further prevents some men from seeking help due to the negative experience they encountered from professional services such as counselling. For example, Hogan, Clarke and Ward's (12) study highlighted participants’ views of calling a helpline and receiving a gender-stereotypical treatment, which construes the men as abusers and not the victims, thus leading to their being treated with suspicion. Some participants even indicated feeling worse and embarrassed for speaking up due to the negative experience they encountered, which led them to decide never to seek help (12).

vii Resort to informal platform for support

Several studies have reported that individuals resort to informal platforms, such as family and friends, for support rather than formal channels, with men being less likely to seek support (11, 12, 28, 32). While informal channels are used, they are often considered unhelpful due to being treated with suspicion and the silence they receive, which further leads to a state of confusion and isolation (12). Even when family members appeared to show concern, once they found out it was a woman who inflicted the injury suffered by the male victims, they laughed, further reinforcing the feeling of staying silent as opposed to speaking up or seeking help (12, 32). Again, gendered expectations intersect with a perception characterised by disbelief of the likelihood of males as victims of gender-based violence, thus leading to marginalisation in support rendered to the affected population.

viii Psychosocial interventions

Several studies reported that men may resort to psychosocial interventions such as counselling and therapy; however, most experiences were negative, perceived as unhelpful due to being disbelieved and also receiving poor support (12). One study reported a man who was homeless as a result of gender-based violence, went to the council and was precluded from ticking the box indicating fleeing domestic violence as it was only for women (12). As a result, studies have highlighted affected victims’ quest for gender-specific interventions related to the need for facts as experienced by the affected victims as opposed to those reflecting traditional values of hegemonic masculinity (12, 32). Another study highlighted males’ preference for speaking with fellow males or females in a safe space without fear of being judged as a preferred mechanism for support (32). One study also indicated that affected male victims often resort to prayer or confide in their religious leaders, such as imams (28).

## Discussion and conclusion

The purpose of this review was to synthesise studies for minoritised men affected by gender-based violence and interventions to improve their psychosocial wellbeing in the UK. In total, 10 studies met the review’s inclusion criteria. To ensure rigour, three reviewers engaged in the risk of bias assessment by evaluating the trustworthiness of individual studies and the overall evidence to identify any systematic errors or biases in study design, its conduct, or analysis that could distort results. Adopting the risk of bias for each study (see Appendix) ensured transparency and helped determine confidence in findings for better decision-making in practice and guidelines as appropriate. While our systematic review highlights a significant gap in the literature on Black, Asian and Minority Ethnic (BAME) men’s experiences of gender-based violence, help-seeking behaviours and interventions to improve their psychosocial wellbeing, the synthesised study yielded several key findings. These include the myriad forms of abuse experienced, masculinity and gendered perceptions of men as abusers as barriers to help-seeking.

A dominant theme found in the review was the prevalence of sexual and physical abuse affecting males’ experience, and this was reported in seven out of the 10 studies (11, 26–31). As such, a significant finding from the review is that, while previous literature emphasised abuse experienced by females (33, 34), minoritised men also face myriad forms of abuse, including sexual (26, 27), physical, and emotional abuse (11, 31). The impact of this abuse is multifaceted and often tends to predict the likelihood of experiencing further abuse as they grow older. Such a feeling of enduring abuse, as our review finds, is further made worse by gendered expectations of men as the head and less likely to be a victim of abuse (12, 24, 32, 48). As such, men are characterised as the abusers. Highlighting the intersection of societal expectations that create a negative gendered stereotype of men, coupled with the abuse that minoritised men face. The implication is that it limits their help-seeking behaviours and leads to adverse outcomes such as feelings of guilt and the tendency to suffer in silence.

Another notable finding is the perceived impact of gender-based violence on minoritised men which six out of the 10 studies indicated in the theme on psychological distress to adversely impact this population (11, 12, 24, 28, 31, 32). The impact as reported in our review includes post-traumatic stress disorders (12, 24, 28, 31), depression, anxiety, and, in its extreme, suicidal ideation (12). While this remains a significant challenge, there is a limited culturally appropriate intervention to support minoritised men affected by gender-based violence in the UK. As our review shows, four out of 10 studies reported using informal sources such as friends and family, which were found to be largely counterproductive (11, 12, 28, 32). Only three of 10 studies reported the use of psychosocial interventions (12, 28, 32). This intervention includes counselling, therapy, and seeking support from religious leaders such as imams (28). The implication of the findings is that it paints a concerning picture of significant unmet needs for the affected population, and disproportionate emphasis is accorded to the affected victims compared to their female counterparts. Of which, females affected by gender-based violence with psychological needs received a range of interventions, including counselling (35, 36), school-based intervention (37, 38), and informal support to help alleviate their suffering (23, 39). Thus, calling for an urgent need for psychosocial intervention for minoritised males.

Finally, themes that emanated from the synthesised studies reveal that several factors limit help-seeking, and these include masculinity, societal perceptions of men as abusers, religio-cultural factors, and victimisation from service providers as barriers to help-seeking. Concerning masculinity, five out of the 10 studies emphasised on how societal expectation of men could serve to constrain their ability to seek help—thereby triggering a feeling of shame to be classed as a victim of abuse (11, 12, 24, 28, 32). The findings are consistent with previous research in countries such as Nigeria (40) and the United States (41) on societal expectations of males as the head of the family, as strong and not susceptible to vulnerabilities.

Regarding societal perceptions of men as abusers, four out of the 10 studies emphasised the gendered stereotype of abuse, which adversely affects males who are often construed as the perpetrator (11, 12, 24, 32). The findings allude to previous literature on men as dominant offenders (42). A plausible explanation for our findings could also be attributed to the overrepresentation of men in prisons compared to women. For example, in the United Kingdom, as of March 2024, males made up 95.9% of the total prison population, while females made up 4.1% (43). Another plausible explanation relates to the role of religio and cultural factors in limiting help-seeking. As our review shows, five out of 10 studies cited religio-cultural factors as barriers to help-seeking in various forms, including the reason for not leaving abusive relations and also as the basis for resorting to silence in order to maintain cultural ideals and obligations (11, 12, 24, 28, 32). This represents a unique finding in that BAME males still hold onto cultural norms from the country of origin even while settled in the UK. The findings appear not to be in congruence with other studies in countries like South Africa, which cited other reasons such as fear (44), and in the United States, other studies have cited shame as the reason why men do not seek help (45, 46).

Regarding help-seeking, the review found that 2 of 10 studies suggest that victimisation by service providers further discourages help-seeking. Such victimisation is characterised by unhelpful responses such as being ridiculed or blamed for the abuse experienced (11, 12). A plausible explanation for such experience could be characterised by societal gendered assumptions of males as the perpetrators of abuse, thus further intersecting to negatively impact their help-seeking behaviours. Another unintended victimisation is the lack of support from local authorities for affected victims (12). For example, one male victim reported being unable to access accommodation because it is not available to him as a male (12). Thus, highlighting the intersection of gendered male stereotypes and government policies, which serve to further marginalise males affected by gender-based violence.

A notable limitation of our review was that it did not include randomised controlled trials, nor were we able to conduct a meta-synthesis. The reason for this is that, at the time of the review, no study appears to have used a randomised controlled trial or meta-synthesis. A further limitation of the study is the inclusion of qualitative studies (which are often associated with small sample sizes), thereby limiting generalisability. For example, Hogan, Clarke, and Ward (12, 32), while highlighting significant findings relating to masculinity and perceived shame as factors limiting help-seeking, are limited by their small sample sizes of 26 men each. Another notable limitation is the limited availability of data, with only a few studies reporting ethnicity. This constitutes a limitation, as studies that did not report ethnicity were not included. Future studies are encouraged to embed equality, diversity, and inclusion in their reporting of participants’ or respondents’ demographic information in their study to enable a critical understanding of how gender-based violence affected diverse ethnicities. This is to avoid a one-size-fits-all approach, which can be counterproductive when providing psychosocial support and services to the affected population. There were also issues with reporting specific ethnic outcomes in studies, as BAME tends to be conflated with other ethnic groups, which makes it challenging to disaggregate their specific experiences. As a result, future studies are encouraged to include ethnicity as appropriate, not only in the methodology but also in the analysis.

Notwithstanding the limitations, our review is the first systematic review investigating Black, Asian and Minority Ethnic (BAME) men’s experiences of gender-based violence, help-seeking behaviours and interventions to improve their psychosocial wellbeing in the UK, thus delineating its originality and significant contribution. Another strength is the methodological rigour underpinned by the PICo model and Boolean operators (AND, OR, NOT) in formulating our review strategy. In conclusion, the review points to the significant gap in interventions for BAME males affected by gender-based violence. This is despite the adverse effect it had on their mental health and psychosocial well-being. Thus, our findings make a valuable contribution that could inform interventions to address the challenges faced by BAME males.

### Implications

A practical implication of our review for policy is the need to understand the adverse impact of gender-based violence on BAME men and the urgent need for culturally appropriate intervention for the affected victims. Creating awareness through educating the masses on avoiding harmful stereotypical gendered norms that perceive the male as the abuser in all GBV cases is crucial to addressing poor help-seeking and stigmatising behaviours. Service providers are also encouraged to adopt a friendly, non-judgmental approach when dealing with male victims of gender-based violence.

## Data Availability

The original contributions presented in the study are included in the article/Supplementary material, further inquiries can be directed to the corresponding author.
