# Supplementary material for: Black, Asian and Minority Ethnic men’s experiences of gender-based violence, help-seeking behaviours and psychosocial interventions in the United Kingdom: a systematic review
Source: Front Public Health. 2026 Mar 13;14:1695675. doi: 10.3389/fpubh.2026.1695675 (PMC13023378; doi:10.3389/fpubh.2026.1695675)
Supplement: Supplementary file 1 [file Table_1.docx]

**Appendix 1: Study Characteristics**

| Author(s) | Aims | Population | Methods | Findings | Strengths | Limitation(s) |
| --- | --- | --- | --- | --- | --- | --- |
| Bates (2019) | The study explored the impact of men’s experiences of intimate partner violence (IPV) in a non-help-seeking sample. | 161 men comprising White (77.6%) with others identifying as having a mixed ethnic background (5.6%), Asian (1.9%), Black (0.6%), Other (2%) or chose not to answer (13%). | Online qualitative survey | The study found that society perceived men who experience abuse as weak and the abusers. Thus, limiting help seeking behaviours. | Comprise men with lived experiences. | There is data missing about validity criteria and coder agreement as the study was a single authored paper and thus could be potentially susceptible to analytical bias. |
| Bebbington et al (2011) | The study aims to establish the sociodemographic distribution of sexual abuse in the general population. | 7,353-household population comprising ethnic minorities. | Quantitative method. | The study found that of the 7,353-household population, 0.8% of men reported child sexual abuse and experiencing same increased the chance of further sexual abuse in adulthood and engagement in prostitution. | Large sample size. | Limited insight to supplement reasons behind the trend associated with child sexual abuse. |
| Cockbain, Ashby and Brayley (2017) | The study explored the gaps in knowledge of boys affected by sexual exploitation. | 9,042 users of child sexual exploitation services comprising Black, Asian and Mixed race. | Quantitative study | More male was known to have criminal records with males also suspected to experience child sexual abuse. | Large dataset. | Underrepresented minoritised population. |
| Gill and Begum (2023) | The study examines how male British South Asian Child Sexual Abuse (CSA) survivors made sense of their experiences. | South Asian CSA male survivors | Qualitative method underpinned by IPA and semi-structured interview | The study found that cultural practices and fear of not believed hindered help seeking behaviour. | Focus on underrepresented population. | Small sample size. |
| Hester et al (2015) | The study measured the experience and perpetration of negative behaviour, including domestic violence and abuse (DVA), and investigate its associations with health conditions and behaviours in men. | 1403 male patient including black and minority ethnic men. | Quantitative method. | 22.7% (95% CI 20.2% to 24.9%) of men reported ever experiencing negative behaviour (feeling frightened, physically hurt, forced sex, ask permission) from a partner. All negative behaviours were associated with a twofold to threefold increased odds of anxiety and depression symptoms in men. | The study constitutes a significant survey of a European clinical population to measure prevalence of negative behaviour and DVA experience and perpetration for male patients. | The 42% of men declining to participate introduces potential bias. The study cross sectional approach limits findings. |
| Hogan, Clarke and Ward (2021) | The study explored men's help-seeking experiences and/or their perceptions of utilising support services/ support networks following IPV victimisation. | White British (19), White Other (5), British Pakistani (1), Black Afro-Caribbean (1) | Qualitative study incorporating semi-structured interviews. | The study found that the importance of maintaining a sense of masculinity including men's fear of being judged negatively pose challenges to help seeking behaviours. | The study highlighted the barriers to help seeking male faced. | The study only focused on men who self-identified as victims of female-perpetrated IPV and might have missed out on the experience of men in same sex relationship with lived experiences of gender-based violence. |
| Hogan, Clarke and Ward (2024) | The study explored masculine ideologies’ impact on the way in which 26 men made sense of their experiences of female perpetrated intimate partner violence (IPV). | White British 19, White Other 5, British Pakistani 1, Black Afro Caribbean 1 | Qualitative method. | The study found that embarrassment and shame for affected male not having met dominant cultural expectations surrounding the roles of men in heterosexual relationships | Incorporate men with lived experiences. | Small sample size and limit basis for generalisability. |
| Jaspal et al., (2024) | The study examined the possible HIV-risk factors in black and minority ethnic men. | Four hundred and thirty-two black and minority ethnic men. | Quantitative study | The study found that that black and minority ethnic men who have sex with men with a history of sexual abuse reported higher frequency of drug use, and of homophobia and racism than those reporting no prior sexual abuse | Include minoritised population. | The study did not include a qualitative result to give further insight into why the result are as they are. |
| Khadr et al., (2018) | The study examined the characteristics of adolescents presenting to sexual assault services and mental and sexual health outcomes after an assault. | The study includes respondents for the black, South Asian and mixed-race population. | Longitudinal study. | The study found that adolescents who presented after sexual assault had very high levels of deprivation and initial vulnerability. | Longitudinal approach. | Limited sample of minoritised men. |
| Myrie & Schwab (2023) | The study explored the lived experience of recovery from Child Sexual Abuse (CSA) among African-Caribbean Black male survivors living in the United Kingdom, Canada, and the United States. | Six participants who identified as Afro-Caribbean (n = 1), Black Caribbean (n= 2), Black Jamaican (n= 2) and South African (n= 1). Four participants were living in Canada, one in the USA, and one in the UK. | Qualitative method underpinned by semi-structured interview and Interpretative phenomenological analysis. | The study found that Black male survivors are surrounded by unique historical/sociocultural interrelationships that impacts their recovery from CSA, including discrimination, institutional racism narratives of masculinity, and other cultural norms. | The study incorporates critical race theory in examining the lived experienced of underrepresented population affected by CSA. | Small sample size. |

**Appendix 2: The Joanna Briggs Institute (JBI) Critical Appraisal Checklist for Qualitative Research**

| Author(s) | 1. Is there congruity between the stated philosophical perspective and the research methodology?  Yes/No/Unclear/ Not applicable | 2. Is there congruity between the research methodology and the research question or objectives? Yes/No/Unclear/ Not applicable | 3. Is there congruity between the research methodology and the methods used to collect data? Yes/No/Unclear/ Not applicable | 4. Is there congruity between the research methodology and the representation and analysis of data? Yes/No/Unclear/ Not applicable | 5. Is there congruity between the research methodology and the interpretation of results? Yes/No/Unclear/ Not applicable | 6. Is there a statement locating the researcher culturally or theoretically? Yes/No/Unclear/ Not applicable | 7. Is the influence of the researcher on the research, and vice- versa, addressed? Yes/No/Unclear/ Not applicable | 8. Are participants, and their voices, adequately represented? Yes/No/Unclear/ Not applicable | 9. Is the research ethical according to current criteria or, for recent studies, and is there evidence of ethical approval by an appropriate body? Yes/No/Unclear/ Not applicable | 10. Do the conclusions drawn in the research report flow from the analysis, or interpretation, of the data? Yes/No/Unclear/ Not applicable | 11. Overall appraisal: include, exclude, seek further info | 12.Comments including reasons for exclusion |
| --- | --- | --- | --- | --- | --- | --- | --- | --- | --- | --- | --- | --- |
| Bates (2019) | Yes | Yes | Yes | Yes | Yes | Yes | Yes | Yes | Yes | Yes | Include | The research focussed on the target population and met the review inclusion criteria. |
| Gill and Begum (2023) | Yes | Yes | Yes | Yes | Yes | Unclear | Unclear | Yes | Yes | Yes | Include | The study was ethically conducted and met the review inclusion criteria. |
| Hogan, Clarke and Ward (2021) | Yes | Yes | Yes | Yes | Yes | Unclear | Yes | Yes | Yes | Yes | Yes | The study included hard to reach population often under-represented in research. |
| Hogan, Clarke and Ward (2024) | Yes | Yes | Yes | Yes | Yes | Unclear | Yes | Yes | Yes | Yes | Yes | The study was robust and met the inclusion criteria. |
| Myrie & Schwab (2023) | Yes | Yes | Yes | Yes | Yes | Yes | Yes | Yes | Yes | Yes | Yes | The study adopts a clear transparent methodology meeting the review’s inclusion criteria. |

**Appendix 3: Quality Appraisal Checklist for Quantitative Studies (Symonds & Tang et al., 2024)**

| Author(s) | 1. Was the study setting (venues) and/or geographic location described in detail? | 2. Were the recruitment processes explained? | 3. Was the administration process explained? | 4. Did the researchers receive informed consent from human participants? | 5. Is there a clear statement about ethical practices? | 6. Was the study sample described in detail? | 7. Were the numbers of people who did/did not consent to participate stated? | 8. Was the number of participants justified? | 9. Was the missingness of data mentioned? | 10. Was the number of participants at each stage/ wave specified? | 11. Did the authors report their missing data handling strategy? | 12. Was the reliability and validity of measurement methods mentioned? | 13.  For studies with repeated measures / longitudinal data |
| --- | --- | --- | --- | --- | --- | --- | --- | --- | --- | --- | --- | --- | --- |
| Bebbington et al (2011) | Yes | Yes | Yes | Yes | Yes | Yes | Yes | Yes | Yes | Yes | Yes | Yes | N/a |
| Cockbain, Ashby and Brayley (2017) | Yes | Yes | Yes | Yes | Yes | Yes | Yes | Yes | Yes | Yes | Yes | Yes | N/a |
| Hester et al (2015) | Yes | Yes | Yes | Yes | Yes | Yes | Yes | Yes | Unclear | Yes | Yes | Yes | N/a |
| Jaspal et al., (2024) | Yes | Yes | Yes | Yes | Yes | Yes | Yes | Yes | Unclear | Yes | Unclear | Yes | N/a |
| Khadr et al., (2018) | Yes | Yes | Yes | Yes | Yes | Yes | Yes | Yes | Yes | Yes | Yes | Yes | N/a |

**Appendix 4: PRISMA Checklist**

| **Section and Topic** | **Item #** | **Checklist item** | **Location where item is reported** |
| --- | --- | --- | --- |
| **TITLE** | | |  |
| Title | 1 | Identify the report as a systematic review. | Title and abstract |
| **ABSTRACT** | | |  |
| Abstract | 2 | See the PRISMA 2020 for Abstracts checklist. | Abstract |
| **INTRODUCTION** | | |  |
| Rationale | 3 | Describe the rationale for the review in the context of existing knowledge. | Introduction |
| Objectives | 4 | Provide an explicit statement of the objective(s) or question(s) the review addresses. | Introduction |
| **METHODS** | | |  |
| Eligibility criteria | 5 | Specify the inclusion and exclusion criteria for the review and how studies were grouped for the syntheses. | Methods |
| Information sources | 6 | Specify all databases, registers, websites, organisations, reference lists and other sources searched or consulted to identify studies. Specify the date when each source was last searched or consulted. | Methods |
| Search strategy | 7 | Present the full search strategies for all databases, registers and websites, including any filters and limits used. | Methods |
| Selection process | 8 | Specify the methods used to decide whether a study met the inclusion criteria of the review, including how many reviewers screened each record and each report retrieved, whether they worked independently, and if applicable, details of automation tools used in the process. | Methods |
| Data collection process | 9 | Specify the methods used to collect data from reports, including how many reviewers collected data from each report, whether they worked independently, any processes for obtaining or confirming data from study investigators, and if applicable, details of automation tools used in the process. | Methods |
| Data items | 10a | List and define all outcomes for which data were sought. Specify whether all results that were compatible with each outcome domain in each study were sought (e.g. for all measures, time points, analyses), and if not, the methods used to decide which results to collect. | Methods |
|  | 10b | List and define all other variables for which data were sought (e.g. participant and intervention characteristics, funding sources). Describe any assumptions made about any missing or unclear information. | Methods and Appendix |
| Study risk of bias assessment | 11 | Specify the methods used to assess risk of bias in the included studies, including details of the tool(s) used, how many reviewers assessed each study and whether they worked independently, and if applicable, details of automation tools used in the process. | Methods and Appendix |
| Effect measures | 12 | Specify for each outcome the effect measure(s) (e.g. risk ratio, mean difference) used in the synthesis or presentation of results. | Not applicable |
| Synthesis methods | 13a | Describe the processes used to decide which studies were eligible for each synthesis (e.g. tabulating the study intervention characteristics and comparing against the planned groups for each synthesis (item #5)). | Methods and appendix |
|  | 13b | Describe any methods required to prepare the data for presentation or synthesis, such as handling of missing summary statistics, or data conversions. | Not applicable |
|  | 13c | Describe any methods used to tabulate or visually display results of individual studies and syntheses. | Methods and appendix |
|  | 13d | Describe any methods used to synthesize results and provide a rationale for the choice(s). If meta-analysis was performed, describe the model(s), method(s) to identify the presence and extent of statistical heterogeneity, and software package(s) used. | Methods and appendix |
|  | 13e | Describe any methods used to explore possible causes of heterogeneity among study results (e.g. subgroup analysis, meta-regression). | Not applicable |
|  | 13f | Describe any sensitivity analyses conducted to assess robustness of the synthesized results. | Methods and appendix |
| Reporting bias assessment | 14 | Describe any methods used to assess risk of bias due to missing results in a synthesis (arising from reporting biases). | Methods and appendix |
| Certainty assessment | 15 | Describe any methods used to assess certainty (or confidence) in the body of evidence for an outcome. | Methods and appendix |
| **RESULTS** | | |  |
| Study selection | 16a | Describe the results of the search and selection process, from the number of records identified in the search to the number of studies included in the review, ideally using a flow diagram. | Methods and appendix |
|  | 16b | Cite studies that might appear to meet the inclusion criteria, but which were excluded, and explain why they were excluded. | Methods |
| Study characteristics | 17 | Cite each included study and present its characteristics. | Methods, findings and appendix |
| Risk of bias in studies | 18 | Present assessments of risk of bias for each included study. | Methods and appendix |
| Results of individual studies | 19 | For all outcomes, present, for each study: (a) summary statistics for each group (where appropriate) and (b) an effect estimate and its precision (e.g. confidence/credible interval), ideally using structured tables or plots. | Not applicable |
| Results of syntheses | 20a | For each synthesis, briefly summarise the characteristics and risk of bias among contributing studies. | Methods and appendix |
|  | 20b | Present results of all statistical syntheses conducted. If meta-analysis was done, present for each the summary estimate and its precision (e.g. confidence/credible interval) and measures of statistical heterogeneity. If comparing groups, describe the direction of the effect. | Findings |
|  | 20c | Present results of all investigations of possible causes of heterogeneity among study results. | Not applicable |
|  | 20d | Present results of all sensitivity analyses conducted to assess the robustness of the synthesized results. | Methods and appendix |
| Reporting biases | 21 | Present assessments of risk of bias due to missing results (arising from reporting biases) for each synthesis assessed. | Methods and appendix |
| Certainty of evidence | 22 | Present assessments of certainty (or confidence) in the body of evidence for each outcome assessed. | Methods and appendix |
| **DISCUSSION** | | |  |
| Discussion | 23a | Provide a general interpretation of the results in the context of other evidence. | Findings and discussion |
|  | 23b | Discuss any limitations of the evidence included in the review. | Discussion |
|  | 23c | Discuss any limitations of the review processes used. | Discussion |
|  | 23d | Discuss implications of the results for practice, policy, and future research. | Discussion |
| **OTHER INFORMATION** | | |  |
| Registration and protocol | 24a | Provide registration information for the review, including register name and registration number, or state that the review was not registered. | Abstract and methods |
|  | 24b | Indicate where the review protocol can be accessed, or state that a protocol was not prepared. | Abstract and methods |
|  | 24c | Describe and explain any amendments to information provided at registration or in the protocol. | Not applicable |
| Support | 25 | Describe sources of financial or non-financial support for the review, and the role of the funders or sponsors in the review. | Not applicable |
| Competing interests | 26 | Declare any competing interests of review authors. | Not applicable |
| Availability of data, code and other materials | 27 | Report which of the following are publicly available and where they can be found: template data collection forms; data extracted from included studies; data used for all analyses; analytic code; any other materials used in the review. | Methods and discussion |
